# Fosgonimeton, a Novel Positive Modulator of the HGF/MET System, Promotes Neurotrophic and Procognitive Effects in Models of Dementia

**DOI:** 10.1007/s13311-022-01325-5

**Published:** 2022-12-20

**Authors:** Jewel L. Johnston, Sherif M. Reda, Sharay E. Setti, Robert W. Taylor, Andrée-Anne Berthiaume, William E. Walker, Wei Wu, Hans J. Moebius, Kevin J. Church

**Affiliations:** Athira Pharma, Inc., 18706 North Creek Parkway, Suite 104, Bothell, WA 98011 USA

**Keywords:** Alzheimer’s dementia, Hepatocyte growth factor (HGF), Neurotrophic factor, Neurodegeneration, Fosgonimeton, ATH-1017

## Abstract

All types of dementia, including Alzheimer’s disease, are debilitating neurodegenerative conditions marked by compromised cognitive function for which there are few effective treatments. Positive modulation of hepatocyte growth factor (HGF)/MET, a critical neurotrophic signaling system, may promote neuronal health and function, thereby addressing neurodegeneration in dementia. Here, we evaluate a series of novel small molecules for their ability to (1) positively modulate HGF/MET activity, (2) induce neurotrophic changes and protect against neurotoxic insults in primary neuron culture, (3) promote anti-inflammatory effects in vitro and in vivo, and (4) reverse cognitive deficits in animal models of dementia. Through screening studies, the compound now known as fosgonimeton-active metabolite (fosgo-AM) was identified by use of immunocytochemistry to be the most potent positive modulator of HGF/MET and was selected for further testing. Primary hippocampal neurons treated with fosgo-AM showed enhanced synaptogenesis and neurite outgrowth, supporting the neurotrophic effects of positive modulators of HGF/MET. Additionally, fosgo-AM protected against neurotoxic insults in primary cortical neuron cultures. In vivo, treatment with fosgo-AM rescued cognitive deficits in the rat scopolamine amnesia model of dementia. Although fosgo-AM demonstrated several procognitive effects in vitro and in vivo, a prodrug strategy was used to enhance the pharmacological properties of fosgo-AM, resulting in the development of fosgonimeton (ATH-1017). The effect of fosgonimeton on cognition was confirmed in a lipopolysaccharide (LPS)–induced neuroinflammatory mouse model of dementia. Together, the results of these studies support the potential of positive modulators of HGF/MET to be used as novel therapeutics and suggest the drug candidate fosgonimeton might protect against neurodegeneration and be therapeutic in the management of Alzheimer’s disease and other types of dementia.

**Supplementary Information:**

The online version contains supplementary material available at 10.1007/s13311-022-01325-5.

## Introduction

An estimated 57 million people worldwide have been diagnosed with dementia [1]. This number is expected to surpass 150 million by the year 2050 as the global population ages [1]. In the USA alone, the predicted cost of care in 2022 for those with dementia is $321 billion, with an additional estimated cost of $271.6 billion in unpaid care provided by family caregivers [2]. The term dementia covers a range of various disorders characterized by substantial cognitive impairment that interferes with regular cognitive function [3]. Alzheimer’s disease (AD) is the most common form of dementia, representing between 60 and 80% of all cases, and is biologically distinguishable from other forms of dementia by accumulation of the neurotoxic proteins beta-amyloid and pathological tau [2]. Other common types of dementia include vascular cognitive impairment, marked by the presence of cerebrovascular disease; α-synuclein diseases, including Lewy body disease and Parkinson’s disease dementia; frontotemporal lobe dementia; and mixed dementias displaying multiple conditions [2].

Despite the varied etiologies of dementia, many pathological processes are shared in cognitive impairment, including neurodegeneration, reduced neuroplasticity, mitochondrial stress, excitotoxicity, oxidative stress, cholinergic deficits, and neuroinflammatory changes [4–6]. The ideal therapy for the management of AD and other types of dementia should target multiple aspects of the disease. The approved therapeutic agents for the management of AD are mainly designed to achieve 1 of 2 goals: to reduce symptoms of the disease (acetylcholinesterase inhibitors and an *N*-methyl-d-aspartate [NMDA] receptor antagonist) or to slow disease progression (anti–amyloid beta immunotherapies) [6, 7]. Given that relatively few therapies are approved and because of the complex pathological processes of AD and other types of dementia, alternative approaches that are multimodal and target neurodegeneration, neuroinflammation, and other contributing factors should be investigated to address this critical unmet need. The need is well-recognized in the field, which is reflected in the multitude of clinical candidates targeting these varied pathological components [8, 9].

Based on evidence from the literature, neurotrophic factors could be used as a multifaceted approach to the management of AD and other types of dementia. Neurotrophic factors mediate neural development, neurogenesis, neuroprotection, synaptogenesis, and immunomodulation [10]. Hepatocyte growth factor (HGF) is a potent neurotrophic factor active in many cell types of the central nervous system (CNS) [11]. HGF activity, through the receptor tyrosine kinase MET, promotes survival and regeneration in a variety of neuron types, including hippocampal, midbrain dopaminergic, cortical, motor, sensory, and cerebellar granular neurons [11]. HGF signaling is also active in glial cells [12]. Activation of the HGF/MET system induces a variety of proneuronal and procognitive processes, and the neurophysiological effects of HGF are well-suited to promote neuronal survival and potentially address the neurodegenerative cascade observed in AD and other types of dementia [11, 13, 14]. The potential of HGF-targeted treatments has been tested both in vitro and in vivo. When exogenously applied in vitro, HGF enhances synaptic long-term potentiation (LTP) in the CA1 region of the hippocampus [15], and overexpression of HGF in vivo improves memory and learning after cerebral infarction in rats [16]. Augmentation of the HGF/MET system slows disease progression and restores function in rodent models of AD [17], as well as in neurotrauma and other neurodegenerative disorders in other models, including in nonhuman primates [18–22].

Therapeutic use of HGF and other neurotrophic factors has been hampered by limitations in drug delivery. Here, we identify small-molecule compounds that positively modulate the HGF/MET system, and evaluate the neuroactivity of a highly potent compound in vitro and in vivo, subsequently optimizing its drug-like characteristics. This compound, fosgo-AM (previously ATH-1001), the active metabolite of fosgonimeton (previously ATH-1017), demonstrated the ability to enhance the HGF/MET system, promote neuroplasticity, and protect against several neurotoxic insults. In vivo, fosgo-AM overcame cholinergic signaling deficits, possibly by enhancing synaptic function and NMDA receptor signaling, and restored cognitive function in the scopolamine amnesia model of cognitive impairment. To improve the drug-like characteristics of fosgo-AM, we developed the prodrug and clinical candidate fosgonimeton, which has enhanced solubility and stability. The procognitive effects of fosgonimeton were confirmed in an animal model of neuroinflammation-induced cognitive impairment. Taken together, these data indicate the promising ability of fosgonimeton to combat several pathological aspects of dementia, including cognitive impairment due to cholinergic signaling deficits, neuroinflammation, reduced neuroplasticity, mitochondrial stress, excitotoxicity, and oxidative stress. Additional studies must be performed to confirm the physiological mechanisms by which fosgonimeton exerts its procognitive effects in vivo.

## Methods

### MET Pathway Activation

#### Phosphorylated MET Receptor Enzyme-Linked Immunosorbent Assay

Human embryonic kidney (HEK293, ATCC CRL-1573) cells were grown at 37 °C, 5% CO_2_ in Dulbecco's Modified Eagle Medium (DMEM) + 10% fetal bovine serum (FBS) in T-75 flasks until approximately 90% confluent and then seeded in polystyrene-treated 6-well plates for at least 8 h in serum-free growth media. Recombinant HGF protein (R&D Systems, Minneapolis, Minnesota) with or without experimental small-molecule treatment cocktails (Jubilant Biosys, Uttar Pradesh, India) were prepared in DMEM + 0.1% FBS and added to wells in triplicate for 15 min. Cells were lysed with 180 μL ice-cold RIPA buffer including a phosphatase inhibitor (PhosphataseArrest, G-Biosciences, St. Louis, Missouri) on ice for 15 min and then cleared by centrifugation at 16,000* g*. Bicinchoninic acid (BCA) assays of lysates were used to normalize protein concentration across samples. Phosphorylated MET receptor (pMET) enzyme-linked immunosorbent assay (ELISA) kit processing was carried out according to the manufacturer’s instructions (Phospho-MET [Tyr1234/1235] Sandwich ELISA Kit, CST #7227C, Cell Signaling Technology, Danvers, Massachusetts). Raw absorbance signals were scaled between subthreshold (1 ng/mL HGF) and saturating (10 ng/mL HGF) controls such that HGF 1 ng/mL = 1 and HGF 10 ng/mL = 10. The statistical difference from subthreshold control was determined by Student’s *t*-test. Comparisons were considered statistically significant when *p* < 0.05.

#### Phosphoactivation of Intracellular Signaling Molecules

To determine phosphoactivation status of the intracellular signaling molecules ERK (Thr202/Tyr204) or AKT (Ser473), we used homogenous time-resolved fluorescence (HTRF) kits to assess phosphorylation status based on the energy transfer between donor and acceptor phospho-ERK (pERK) or phospho-AKT (pAKT) antibodies. HTRF kit processing for pERK (Phospho-ERK [Thr202/Tyr204] cellular Advanced kit, #64AERPEG, Cisbio, Codolet, France) and pAKT (Phospho-AKT [Ser473] cellular kit, #64AKSPEG, Cisbio, Codolet, France) were carried out per manufacturer instructions. In brief, HEK293 cells were grown at 37 °C, 5% CO_2_ in DMEM + 10% FBS until approximately 80% confluent. Cells were seeded in polystyrene-treated 96-well cell culture plates at approximately 50,000 cells/well and cultured overnight in 50 µL serum-free growth media. After 18–24 h of overnight culture, recombinant HGF protein (R&D Systems, Minneapolis, Minnesota) with or without fosgo-AM treatments were prepared in DMEM + 0.1% FBS and incubated at a 1:1 ratio at 37 °C, 5% CO_2_, for 60 min. Next, 50 µL of treatment was added to the wells in triplicate for pERK or quadruplicate for pAKT assessments and incubated at 37 °C, 5% CO_2_ for 15 min. After treatment, the supernatant was removed, and cells were lysed with 1× lysis buffer (prepared per manufacturer’s protocol) for 30 min at room temperature (RT) while shaking. Next, 16 µL of cell lysates were transferred to a low-volume 96-well HTRF plate. pERK or pAKT antibodies were prepared and added per manufacturer’s protocol: 2 µL of Eu^3+^ Cryptate working antibody (donor) solution and 2 µL of d2 working antibody (acceptor) solution were added to each well and incubated overnight at RT. Fluorescence emission at 2 different wavelengths (665 nm and 620 nm) were measured on an Envision plate reader (PerkinElmer, Waltham, Massachusetts), and the HTRF ratio of the acceptor and donor emission signals for each well was calculated as follows: HTRF ratio = (665 nm/620 nm) × 10,000. Data for each group were averaged and presented as mean ± SEM. Data were assessed for normality (Shapiro–Wilk) and homoscedasticity (Brown-Forsythe). Statistical significance was determined by one-way analysis of variance (ANOVA) with Tukey’s multiple comparison post-test. Comparisons were considered statistically significant when *p* < 0.05.

#### MDCK Cell Scatter Behavior Assay

Madin-Darby canine kidney (MDCK, ATCC CCL-34) cells were grown at 37 °C, 5% CO_2_ in Eagle’s Minimum Essential Medium (EMEM) + 10% FBS in T-75 flasks until 80% confluent. Cells were then plated at 1400 cells/well in black-walled, clear-bottom 96-well plates and grown at 37 °C under 5% CO_2_ for 24 h. Growth media were replaced by serum-free media for 2 h before treatment. Treatments containing HGF and fosgo-AM were made in serum-free media and applied in quintuplicates for 24 h at 37 °C under 5% CO_2_. Cells were then fixed in chilled 100% EtOH at 4 °C for 20 min and then stained with wheat germ agglutinin conjugated to CF488 (WGA488, Biotium, 29,022–1, Fremont, California) at 2 mg/mL in phosphate-buffered saline (PBS) for 30 min at RT to visualize whole-cell surfaces. Cultures were then imaged with an iCys instrument (iCyte, Thor Labs, Newton, New Jersey) and analyzed with the ImageJ (National Institutes of Health) particle count feature. Scattering behavior was quantified by normalizing particle counts against total stained area. As MDCK colonies scatter, the total number of particles increases relative to the percentage of imaged area covered by cells. Data were assessed for normality (Shapiro–Wilk) and homoscedasticity (Brown-Forsythe). The statistical difference from HGF alone was determined by one-way ANOVA with Dunnett’s multiple comparison post-test with an alpha value of 0.05 (Prism, GraphPad).

### Neurite Outgrowth and Synaptogenesis

#### Animals

The following experiments were carried out in accordance with the *Guide for the Care and Use of Laboratory Animals* (2011 edition) and approved by the National Institutional Animal Care and Use Committee (IACUC, MD Biosciences Inc., Cambridge, Massachusetts). The studies used brain cells harvested from newborn (0–2 days postnatal) Sprague Dawley rats sourced from Envigo RMS, Israel. The health status of the animals was evaluated upon arrival. Only newborns in good health were used in the study.

#### Primary Cell Culture

On day 0, rat hippocampal neurons from newborn pups were isolated and cultured in brain neuronal culture medium (neurobasal medium supplemented with 5% FBS, 2% B-27 supplement, and 0.003% gentamycin) at 37 °C under 5% CO_2_. At 1 day in vitro (DIV1), cells were transferred to a poly-d-lysine–coated 24-well plate at approximately 10,000 cells/well. Cells were then grown in 3 mL of serum-free medium and treated with the following: 0.1% dimethyl sulfoxide (DMSO; vehicle), 10 nM dihydrotestosterone (positive control), 10 nM fosgo-AM, 1 nM fosgo-AM, 5 ng/ml HGF, or 5 ng/ml HGF + 1 nM fosgo-AM. All treatments contained a final concentration of 0.1% DMSO. For the neurite outgrowth assay, treatment media were refreshed on DIV3, and cells were processed for analysis on DIV4. For the synaptogenesis assay, treatment media were refreshed on DIV3 and DIV6, and cells were processed for analysis on DIV8. Processing for both the neurite outgrowth and the synaptogenesis assays was performed as follows: media were removed, and cells were fixed with 100% methanol for 20 min at RT before methanol was aspirated and each well was washed twice with Dulbecco’s phosphate-buffered saline (DPBS). Cells were permeabilized using 0.02% Triton X-1000 (ThermoFisher, Waltham, Massachusetts) in DPBS for 2 min at RT without shaking, and plates were blocked with 5% bovine serum albumin (BSA) in DPBS for 1 h at RT. Plates were then incubated overnight at 4 °C with gentle shaking with use of beta-III tubulin antibody (ab201740; Abcam, Boston, Massachusetts) to assess neurite outgrowth or with use of 1:500 rabbit anti–synaptobrevin-II antibody (Synaptic Systems, cat# 104 202, Göttingen, Germany) to assess synaptogenesis. After overnight incubation, plates were washed 3 times with DPBS and incubated for 1 h at RT with use of anti–rabbit immunoglobulin G–secondary antibody conjugated to Alexa Fluor 488 (Life Technologies, Carlsbad, California, 1:500). After 5 DPBS washes, coverslips were mounted on slides using fluorescent mounting medium (Golden Bridge International Labs, Bothell, Washington, cat# E19-s). Imaging was performed using the BX43 Olympus microscope driven by the standard “CellSens” software by Olympus. Images were taken under 60 × water-dipping objective using a DP74 camera (Olympus, Center Valley, Pennsylvania). Ten images of single cells from a total of 2 assays were analyzed using NeuroJ plug-in for ImageJ to assess neurite outgrowth, as neurite length and the number of branches per neuron were counted for each cell. For the synaptogenesis assay, synaptic count (number of synapses) and synaptic strength (relative abundance of presynaptic vesicles per synapse as measured by synaptobrevin-II fluorescence intensity) were quantified for each cell. Data were assessed for normality (Shapiro–Wilk) and homoscedasticity (Brown-Forsythe). For data analysis, the average of each treatment group was compared with the average of the vehicle (0.1% DMSO) group and analyzed using one-way ANOVA/Dunnett’s post-test or Kruskal–Wallis/Dunn’s post-test (when data did not follow a normal distribution) (Prism, GraphPad). A value of *p* < 0.05 was considered to represent a statistically significant difference.

### Neuroprotection

#### Animals

All animal experimental procedures were approved by the Institutional Animal Ethics Committee (IAEC, Sai Life Sciences Limited, Hyderabad) and strictly carried out in accordance with the guidelines of Committee for the Purpose of Control and Supervision of Experiments on Animals (CPCSEA), Government of India. Every effort was made to minimize the number of animals and their suffering during experimental procedures, including humane euthanasia. The experiment used brain cells harvested from newborn rat pups (1–3 days postnatal) from pregnant Sprague Dawley rats (18–20 days gustatory dams; age 12–14 weeks; weight 275–350 g) procured from Vivo Biotech Limited, Hyderabad, India. Delivered rat pups were observed daily for health status until use in the study; only newborn pups in good health were used for the study.

#### Cell Viability Measurements

A Cell Titer-Glo (CTG) Luminescent Cell Viability Assay (Promega, Cat #G7571, Madison, Wisconsin) was used to assess the neuroprotective potential of fosgo-AM. Brain cortices were dissected from P1–3 rat pups and homogenized in DMEM containing 10% FBS. The resulting suspension was then incubated with a DNase/papain/trypsin solution for 30 min at 37 °C, followed by incubation in a collagenase solution for 1 h. Cells were then seeded at 5000 cells/well in 384-well plates and maintained at 37 °C and 5% CO_2_ in Complete Neurobasal Medium supplemented with B27/GDNF/BDNF containing 10% FBS. One day later, cultures were switched to serum-free neurobasal medium supplemented with 2% B27 and maintained until neuronal maturity (35–40 DIV). Media were changed every 3 days, and fresh medium was applied 24 h before each experiment. Cells were then treated with fosgo-AM at 1 µM, 100 nM, 10 nM, or 1 nM in a 0.5% DMSO vehicle for 15 min, and subjected to several conditional insults, including 1-methyl-4-phenylpryidinium (MPP+; 500 µM), glutamate (25 µM), lipopolysaccharide (LPS; 1 µM), or hydrogen peroxide (H_2_O_2_; 1 µM) for 24 h. After 24 h, 25 µL CTG reagent was added to each well and the plate was incubated with shaking for 15 min. Luminescence measurements to assess relative levels of ATP were performed on an Envision plate reader (PerkinElmer, Waltham, Massachusetts). Data for the vehicle control were set to 100% cell viability, and data for each other group were averaged, normalized to vehicle control, and presented as mean ± SEM. Data were assessed for normality (Shapiro–Wilk) and homoscedasticity (Brown-Forsythe). Statistical analyses were performed using one-way ANOVA with Tukey’s Multiple Comparison test (Prism GraphPad, San Diego, California). A value of *p* < 0.05 was considered to represent a statistically significant difference.

### Scopolamine Morris Water Maze Study

#### Animals

Animals were maintained in accordance with the National Institute of Health Guide for the Care and Use of Laboratory Animals. Rats (14-week-old male Sprague Dawley) from Simonsen laboratories (Gilroy, CA) were used in the scopolamine Morris water maze study. This study was performed at Washington State University and procedures were approved by the Washington State University Institutional Animal Care and Use Committee.

All animals were acclimated to the environment, examined, handled, and weighed before initiation of each study to ensure adequate health and to minimize nonspecific stress-associated problems. During the studies, 12:12-h light–dark cycles were maintained. The RT was maintained between 20 and 23 °C with a constant relative humidity of 50%. Food and water were provided ad libitum for the duration of the studies.

#### Intracerebroventricular Cannulation Surgery

Animals were anesthetized with ketamine hydrochloride plus xylazine (70 mg/kg and 7 mg/kg intraperitoneally, respectively). An intracerebroventricular (ICV) guide cannula (PE-60; Clay Adams, Parsippany, NY) was stereotaxically positioned (model 900; David Kopf Instruments, Tujunga, CA) in the right hemisphere of the brain 1.0 mm posterior and 1.5 mm lateral to the bregma. The guide cannula measured 1.5 cm in length with a heat bulge at 2.5 mm from the beveled tip to control insertion depth. Once in position, the cannula was secured to the skull with screws and dental cement. Animals were housed individually and hand-gentled for 6 days of postsurgical recovery. Histologic verification of cannula placement was accomplished by ICV injection of 4 μL cresyl violet after completion of behavioral testing. Animals for which cannulas were improperly placed were removed from the study.

#### Morris Water Maze Assay

The Morris water maze consisted of a 183-cm diameter, 64-cm deep tank with a clear circular platform (16-cm diameter) placed 30 cm from the maze wall and submerged 1 cm below the surface of the 26.7 °C water. The maze was virtually divided into 4 equal quadrants with the platform centered in 1 quadrant. Eight visual cues were randomly placed on the maze wall and these cues remained consistent throughout the trials.

Animals received either fosgo-AM (1 mg/kg in DMSO) or volume-matched vehicle (DMSO) by subcutaneous (SC) injection 45 min before testing, followed by ICV injection of scopolamine hydrobromide (105 nmol in 4 μL artificial cerebrospinal fluid [aCSF] over a duration of 40 s) 20 min before testing. An investigator blinded to treatment placed the rats on the platform for 30 s before the first trial to allow rats to orient to the platform relative to the extramaze cues. Each animal was subjected to 5 trials per day. For each trial, rats were placed in a pseudo randomly assigned entry quadrant such that entry points differed in subsequent trials. Rats were allowed a maximum of 120 s to find the platform. Animals that did not complete the task within 120 s were guided to the platform. All animals were given 30-s rest and memory consolidation periods on the platform between each of the 5 daily trials. The training/acquisition period ran for a total of 8 consecutive days.

Probe trials were conducted on day 9 to determine learning persistence. In these trials, the platform was removed and a “platform region,” a circular region centered around the previous location of the platform but with twice the original diameter, was virtually established. The percentage of trial time spent within the platform region, the number of times that the animal crossed into the platform region, and the average distance from the center of the platform region were recorded. The swimming patterns of the rats were recorded using video tracking software (MediaRecorder and EthoVision software, Noldus, VA, USA).

#### Statistics

The daily escape latency (defined as the mean latency to find the platform each day) was calculated for each animal. A two-way ANOVA with repeated measures (sphericity was not assumed, Greenhouse–Geisser’s correction was applied) was used to determine significance compared to scopolamine + vehicle controls. Data collected during the probe trials were assessed for normality (Shapiro–Wilk) and homoscedasticity (Brown-Forsythe) and subsequently analyzed using one-way ANOVA with Bonferroni post hoc tests compared with scopolamine + vehicle controls. A value of *p* < 0.05 was considered to represent a statistically significant difference. Numbers were as follows in the control group (8), the scopolamine group (9), and the scopolamine + fosgo-AM group (10).

### Simulated Intestinal and Gastric Fluid Stability

Simulated intestinal and gastric fluid studies were contracted to Quintara Discovery Inc, Hayward, California. Simulated intestinal fluid was freshly prepared with 8.7 mM NaOH, 28.65 mM NaH_2_PO_4_, 5.85 mM NaCl, with a final pH of 6.8. Pepsin was added to a final concentration of 0.32%. Simulated gastric fluid was prepared with 34.2 mM NaCl. The pH was adjusted to 1.2, and pancreatin was added to a final concentration of 1%. Fosgo-AM was added to the simulated fluids to achieve a concentration of 5 µM. After mixing, samples were transferred to 96-well plates in duplicate (25 μL/well) and incubated at 37 °C. At time 0, 1, 2, and 4 h, 150 μL quench solution (100% acetonitrile with 0.1% formic acid) and bucetin internal standard was added to the appropriate wells. Plates were sealed and centrifuged at 4 °C for 15 min at 4000 rpm. The supernatant was transferred to fresh plates for liquid chromatography/dual mass spectrometry (LC/MS/MS) analysis. The extent of metabolism was calculated as the disappearance of the test compound compared with the time = 0 incubations.

### Solubility

#### Fosgo-AM Solubility

A fosgo-AM dilution series was produced in DMSO. Compound stocks were added in triplicate to a 96-well plate containing PBS, resulting in 1% DMSO concentration, and were incubated at 37 °C for 2 h. Turbidity in the sample wells was determined by absorbance at 620 nm. A concentration that resulted in greater than 10% change in absorbance from blank wells was considered insoluble.

#### Fosgonimeton Solubility

Fosgonimeton solubility studies were contracted to GVK Biosciences, Hyderabad, India. Stocks of fosgonimeton were prepared in duplicate at several concentrations in osmolarity-corrected PBS and were vortexed to dissolve into solution. The solutions were filtered through a 0.45-µm filter followed by a 0.22-µm filter to remove insoluble particulates. Pre- and postfiltration aliquots were collected, and their concentration was determined via high-performance liquid chromatography.

### Pharmacokinetic Studies

#### Animals

The fosgonimeton pharmacokinetic (PK) and distribution studies were performed in Sprague Dawley rats bred and maintained in house at GVK Biosciences Hyderabad, India. All studies at GVK (an AAALAC- and CPCSEA-accredited facility) were approved by the GVK Institutional Animal Ethics Committee. Additional PK studies for fosgonimeton were conducted in BALBc mice at Jubilant Biosys, Bengaluru, India (AAALAC and CPCSEA accredited), with protocols approved by the Jubilant Institutional Animal Ethics Committee. Mouse tissue-distribution studies were conducted in C57BL/6 mice (Global, India) at Sai Life Sciences Ltd. Telangana, India, (an AAALAC- and CPCSEA-accredited facility) and approved by the Sai Institutional Animal Ethics Committee. All studies followed the National Institute of Health Guide for the Care and Use of Laboratory Animals.

#### Fosgo-AM PK in Rats

Four male rats were treated with 10 mg/kg fosgo-AM in DMSO via SC injection. Blood was serially collected at 0.08, 0.25, 0.5, 1, 2, and 4 h after dosing. Plasma was obtained by centrifugation with 2% disodium ethylenediaminetetraacetic acid (EDTA) as an anticoagulant. Plasma samples were precipitated with acetonitrile containing internal standard, and the concentration of fosgo-AM was determined by LC/MS/MS.

#### Fosgonimeton PK in Rats

This study was conducted as described in the “Fosgo-AM PK in Rats” section with the following changes: animals were given 12 mg/kg fosgonimeton in saline with 0.1% glycine via SC injection, and a dose-linear correction was applied to normalize the dose to 10 mg/kg for comparison with the fosgo-AM study. Sample concentrations of both fosgonimeton and fosgo-AM were determined.

#### Fosgonimeton PK in Mice

Three male BALBc mice were treated with 0.75 mg/kg fosgonimeton in saline with 0.1% glycine via SC injection. Blood was serially collected at 0.25, 0.5, 1, 2, and 4 h after dosing. Plasma was obtained by centrifugation with 2% disodium EDTA as an anticoagulant. Plasma samples were precipitated with acetonitrile containing internal standard, and the concentration of fosgonimeton and fosgo-AM was determined by LC/MS/MS.

#### Fosgonimeton CNS Distribution in Rats

Three male Sprague Dawley rats per time point were given 12 mg/kg fosgonimeton via SC injection. At 0.16, 0.5, and 1 h after dosing, animals were humanely killed and perfused with chilled saline. Brains were collected and dissected into the following brain regions: whole brain, striatum, olfactory bulb, hippocampus, cerebellum, cerebral cortex, and brainstem. Brain regions were homogenized and the exposures of fosgonimeton and fosgo-AM were determined by LC/MS/MS.

#### Fosgonimeton CNS Distribution in Mice

Four C57BL/6 mice—2 males and 2 females—were treated with 5 mg/kg fosgonimeton via intravenous (IV) injection. At 0.16 h after dosing, animals were humanely killed and perfused with normal saline. The whole brain was collected and homogenized before determination of fosgonimeton and fosgo-AM tissue penetration by LC/MS/MS.

### Neuroinflammation

#### LPS-Induced Cognitive Impairment: Animals

All animal manipulations were conducted in accordance with the European Directive 2010/63/UE published in the French decree 2013–118 of February 1, 2013, and approved by an independent government-accredited ethics committee (CEEA 35, NEUROFIT, France). Animals for the study consisted of 90 four- to five-week-old CD-1 mice (Janvier, Le Genest St Isle, France). Mice were group-housed and maintained in a room with controlled temperature (21–22 °C) and reversed 12/12 light/dark cycle. The animals had access to food and water ad libitum*.*

#### LPS-Induced Cognitive Impairment: Experimental Paradigm

Mice were sorted into the following experimental groups: saline + vehicle (control), LPS + vehicle (LPS), LPS + 0.1 mg/kg memantine, and LPS + fosgonimeton at 0.125, 0.25, 0.5, 1, or 1.25 mg/kg (LPS + fosgo). On day 0 of the study, all groups received either a single intraperitoneal injection of saline or LPS (0.25 mg/kg). During days 1–14, animals received memantine or fosgonimeton treatment once daily. On day 14, 60 min after the last dosing with test compounds or vehicle, mice were assessed for performance on the T-maze spontaneous alternation task.

#### LPS-Induced Cognitive Impairment: T Maze

The T-maze apparatus was made of gray Plexiglas with a main stem (55 cm × 10 cm × 20 cm) and 2 arms (30 cm × 10 cm × 20 cm) positioned at a 90° angle relative to the main stem. A start box (15 cm × 10 cm) was separated from the main stem by a guillotine door. Horizontal doors were also provided to close specific arms during the “forced-choice” alternation task.

The experimental protocol consisted of a single session, which started with 1 forced-choice trial, followed by 14 “free-choice” trials. In the forced-choice trial, the animal was confined for 5 s in the start arm and was then released while either the left or the right goal arm was blocked by a horizontal door. Animals were allowed to explore the maze, eventually entering the open goal arm and afterward returning to the start position. Immediately after the animal returned to the start position, the previously blocked goal door was opened, and the animal was allowed to choose freely between the left and the right goal arm (“free choice trials”). The animal was considered to have entered an arm if it placed all 4 paws in the arm. The session was terminated and the animal was removed from the maze after 14 free-choice trials were performed or 10 min elapsed, whichever occurred first. The apparatus was cleaned between each animal run using 70% ethanol. During the trials, animal handling and the visibility of the operator were controlled to minimize interference during testing.

#### LPS-Induced Cognitive Impairment: Statistical Analysis

The percentage of alternations over the 14 free-choice trials was determined for each mouse and was used as an index of working memory performance. This percentage was defined as entry into a different goal arm of the T maze over successive trials (e.g., left–right–left–right) and was calculated using the formula:

$$\%\; \text{Alternations}=\frac{\text{Number of Alternations}}{14}\times 100$$

Additionally, percentage of recovery was calculated by normalizing the control group to 100% and the LPS group to 0% and calculating the respective relative response for each individual animal, as follows:

$$\begin {aligned}\%\ &\mathrm{Recovery}\\&=\frac{(\mathrm{Individual}\;\%\;\mathrm{alternation}-\mathrm{mean}\;\mathrm{LPS}\;\%\;\mathrm{alternation})}{(\mathrm{Mean}\;\mathrm{control}\;\%\;\mathrm{alternation}-\mathrm{mean}\;\mathrm{LPS}\;\%\;\mathrm{alternation})}\end {aligned}$$

Data were assessed for normality (Shapiro–Wilk) and homoscedasticity (Brown-Forsythe). One-way ANOVA was performed on the data for spontaneous alternations. Dunnett multiple comparison test was used for post hoc pairwise comparisons with the LPS group. The Kruskal–Wallis test was used for percentage of recovery with Dunn’s multiple comparison post hoc test. A value of *p* < 0.05 was considered to represent a statistically significant difference.

#### Quantification of Proinflammatory Cytokine Release in LPS-Challenged THP-1 Macrophages

To determine the effect of fosgo-AM on LPS-induced cytokine release, we sought to determine the levels of interleukin-1β (IL-1β), interleukin-6 (IL-6), and tumor necrosis factor-α (TNF-α) in LPS-challenged THP-1–differentiated macrophages in the presence and absence of fosgo-AM. THP-1 monocytes were grown at 37 °C, 5% CO_2_ in Roswell Park Memorial Institute (RPMI) medium + 10% FBS until approximately 80% confluent. Cells were seeded in polystyrene-treated 96-well cell culture plates at approximately 50,000 cells/well in 100-µL complete media. 100 µL Phorbol myristate acetate was then added to each well for a final concentration of 100 ng/mL. The plate was then incubated for 3 days at 37 °C, 5% CO_2_ to prime the monocytes into macrophages. Before treatment, the complete media were exchanged for serum-free media and incubated at 37 °C, 5% CO_2_ for 3 h. The media were aspirated, and cells were treated with 50 µL vehicle (0.05% DMSO, 0.05% FBS, 0.5 ng/ml HGF) or fosgo-AM (1 µM, 10 nM, 100 pM, 1 pM or 0.01 pM in vehicle) in triplicate and incubated at 37 °C, 5% CO_2_ for 20 min. After 20 min, the treatment was aspirated and 100 µL LPS (50 ng/mL) was applied to the culture and incubated at 37 °C, 5% CO_2_ for 24 h. For cytokine quantification, cell culture supernatant was assayed by HTRF kits to assess levels of IL-1β (Human IL-1β kit, #62HIL1BPEG, Cisbio, Codolet, France) and IL-6 (Human IL-6 kit, 62HIL06PET, Cisbio, Codolet, France), and by ELISA (Human TNF-α ELISA kit, KHC3011, ThermoFisher, Waltham, Massachusetts) to determine the levels of TNF-α. After 24 h of LPS incubation, 16 µL supernatant or concentration standards were transferred to a low-volume 384-well plate and assayed for IL-1β or IL-6. Appropriate antibodies were prepared and added per manufacturer’s protocol: 2 µL of Eu^3+^ Cryptate working antibody solution (donor) and 2 µL of XL working antibody solution (acceptor) were added to each well and incubated overnight at RT with agitation. Fluorescence emission at 2 different wavelengths (665 nm and 620 nm) were measured on an Envision plate reader (PerkinElmer, Waltham, Massachusetts), and HTRF ratio of the acceptor and donor emission signals for each well was calculated as follows: HTRF ratio = (665 nm/620 nm) × 10,000. The concentration of IL-1β (in picograms per milliliter) and IL-6 (in picograms per milliliter) was calculated from a standard curve generated from the manufacturer’s protocol. For TNF-α, 50 µL cell culture supernatant or TNF-α concentration standards were transferred to a TNF-α antibody–coated 96-well ELISA plate. Standard diluent buffer 50 µL was added to each well, and the plate was incubated for 2 h at RT. The solution in each well was then aspirated and the wells were washed 4 times with wash buffer. TNF-α Biotin conjugate solution 100 µL was added to each well and the plate was incubated for 1 h at RT. After 4 washes, 1 × Streptavidin-HRP solution 100 µL was added to each well and the plate was incubated for 1 h at RT. After 4 washes, Stabilized Chromagen 100 µL was added to each well and the plate was incubated for 30 min at RT. The reaction was stopped with use of Stop solution in each well, and absorbance (450 nm) was measured on a Synergy 2 plate reader (BioTek, Santa Clara, California). The concentration of TNF-α (in picograms per milliliter) was calculated from a standard curve generated from the manufacturer’s protocol.

Data for each group were averaged and presented as mean ± SEM. Data were assessed for normality (Shapiro–Wilk) and homoscedasticity (Brown-Forsythe). Statistical significance was determined by one-way ANOVA with Dunnett’s multiple comparison post-test. Comparisons were considered statistically significant when *p* < 0.05.

## Results

### Identification of Small-Molecule Positive Modulators of the HGF/MET System

**Fig. 1:**
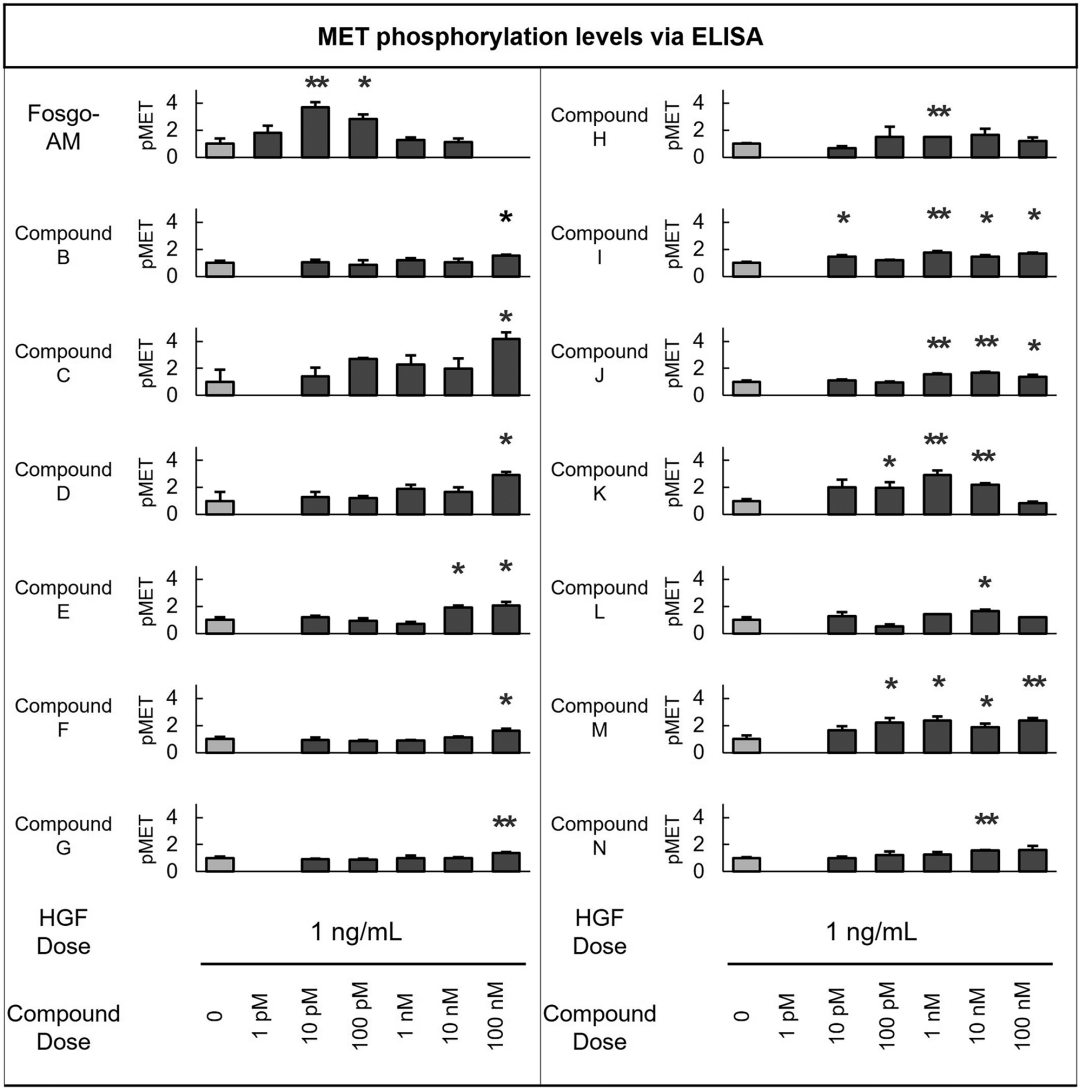
Biochemical identification of positive modulators of HGF/MET. A series of related small-molecule compounds, including the active metabolite of fosgonimeton (fosgo-AM), increase MET receptor phosphorylation (pMET) in the presence of low levels of hepatocyte growth factor (HGF) as assessed by pMET enzyme-linked immunosorbent assay (ELISA) of human embryonic kidney (HEK293) cell lysates. All compounds are added to cells in combination with 1 ng/mL HGF. Data shown are the mean of 3 wells per experimental dose. pMET quantification data for each experiment was scaled between HGF 1 ng/mL = 1 and HGF 10 ng/mL = 10 using controls included in each experiment (HGF standards at 0, 1, 10 ng/mL). Statistical significance was determined for each compound versus HGF alone (1 ng/mL) (*p < 0.05, **p < 0.01, Student’s *t*-test). Missing bars indicate an untested concentration

The MET receptor becomes phosphorylated upon activation by HGF binding [23]. Therefore, an ELISA that detects pMET was used to evaluate the ability of the experimental small molecules to enhance pMET levels in the presence of low concentrations of HGF. Lysates of HEK293 cells treated with subthreshold (1 ng/mL) concentrations of recombinant HGF in combination with a dose range (0.001–100 nM) of 14 experimental small molecules (fosgo-AM and compounds B–N) were analyzed. Several molecules able to enhance pMET levels in the presence of low concentrations of HGF were identified. The most potent positive modulator of HGF/MET was fosgo-AM (Fig. 1).

**Fig. 2:**
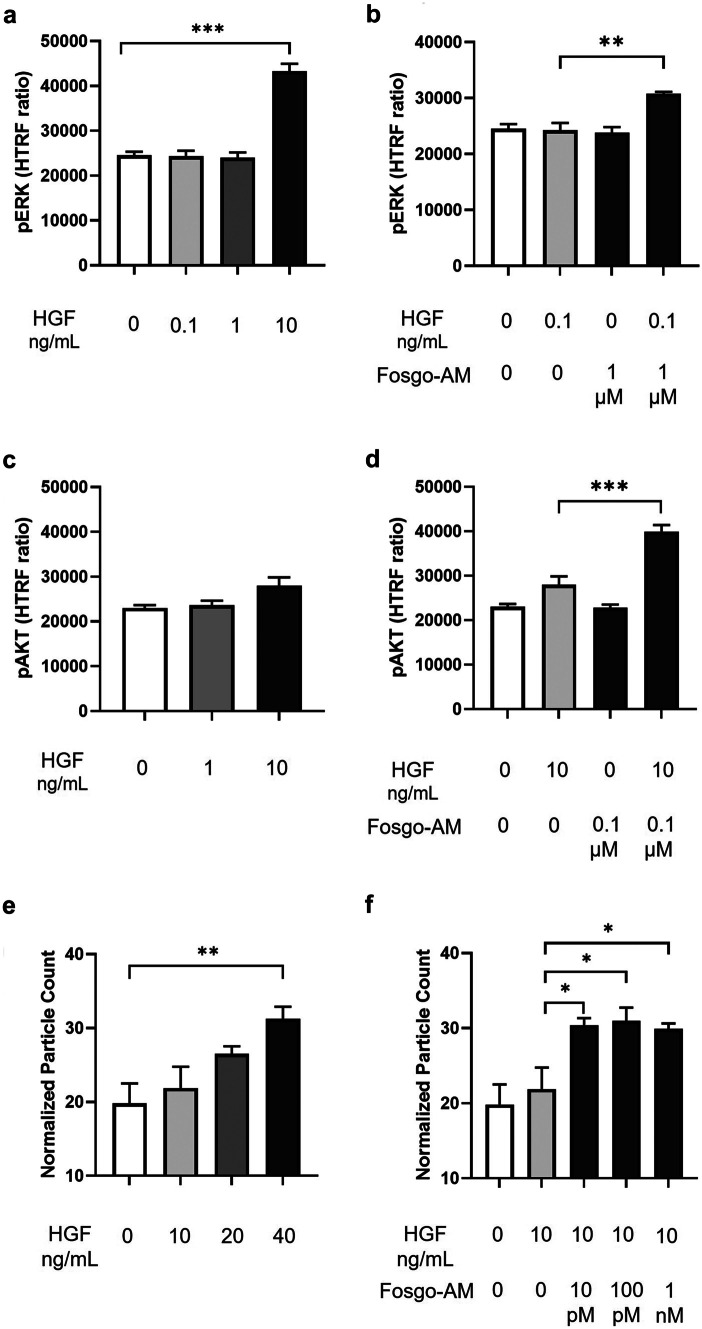
Fosgonimeton active metabolite (fosgo-AM) enhances phosphoactivation of ERK (pERK) and AKT (pAKT) and increases HGF/MET-dependent cell scattering behavior. **a**–**d** Human embryonic kidney (HEK293) cells were treated with hepatocyte growth factor (HGF) ± fosgo-AM, and the corresponding cell lysates were incubated with homogenous time-resolved fluorescence (HTRF) pERK or pAKT specific donor–acceptor antibody pairs to measure relative phosphorylation levels of endogenous ERK or AKT. **a** pERK quantification in the presence of HGF (0–10 ng/mL). HGF (10 ng/mL) significantly increased pERK versus no HGF treatment. **b** pERK quantification in the presence of subthreshold HGF (0.1 ng/mL) ± fosgo-AM (1 µM). Fosgo-AM significantly increased pERK in the presence of subthreshold HGF versus HGF alone. **c** pAKT quantification in the presence of HGF (0–10 ng/mL). **d** pAKT in the presence of subthreshold HGF (10 ng/mL) ± fosgo-AM (0.1 µM). Fosgo-AM significantly increased pAKT in the presence of subthreshold HGF versus HGF alone. Data presented as mean ± SEM; *n* = 3 and 4 for pERK and pAKT, respectively. Statistical differences were determined by one-way ANOVA followed by Tukey’s multiple comparison post-test. **p < 0.01, ***p < 0.001 versus HGF alone. **e**–**f** Madin-Darby canine kidney (MDCK) cells were treated with HGF ± fosgo-AM, stained with a nonspecific surface fluorescent stain (wheat germ agglutinin conjugated to CF488), and imaged to visualize scattering behavior. Data shown are particle counts normalized against total stained area. **e** Quantification of MDCK cell scattering behavior in the presence of HGF (0–40 ng/mL). HGF (40 ng/mL) significantly increased cell scattering behavior. **f** Quantification of MDCK cell scattering behavior in the presence of subthreshold HGF (10 ng/mL) ± fosgo-AM (0.01–1 nM). Data presented as mean ± SEM; *n* = 5. Statistical differences were determined by one-way ANOVA followed by Dunnett’s multiple comparison post-test. *p < 0.05, **p < 0.01

Activation of MET stimulates a variety of intracellular signaling cascades, including phosphoactivation of ERK and AKT, which mediate HGF-dependent cell behaviors and neurotrophic signaling [11]. Therefore, we sought to assess whether fosgo-AM treatment enhances HGF/MET-mediated phosphorylation of ERK and AKT in HEK293 cells. Activation of ERK and AKT was evaluated by HTRF after 15 min of exposure to HGF ± fosgo-AM (Fig. 2). The sensitivity of the assays to HGF was first assessed using a range of HGF doses (0*–*10 ng/mL) (Fig. 2a, c). A subthreshold dose was chosen for subsequent fosgo-AM evaluations. Owing to differential sensitivity of the assays to HGF, 0.1 ng/mL and 10 ng/mL HGF were selected for the pERK and the pAKT assay, respectively. A one-way ANOVA indicated a significant treatment effect on pERK (*F*(2,6) = 16.82, *p* = 0.0035) and pAKT (*F*(2,9) = 36.70, *p* < 0.001). Post hoc multiple comparisons indicated that HEK293 cells treated with fosgo-AM + HGF exhibited a significant increase in levels of pERK (*p* < 0.01; Fig. 2b) and pAKT (*p* < 0.001; Fig. 2d) compared with HGF treatment alone, suggesting that fosgo-AM positively modulates HGF-dependent intracellular pathways that facilitate phosphorylation of ERK and AKT.

A cell-scattering assay was used to evaluate whether the effects of positive modulation of the HGF/MET system by fosgo-AM were sufficient to drive changes in MET-dependent cellular behaviors. Colony-forming cells respond to HGF-dependent MET stimulation by scattering from colony partners [24–26]. We tested the capacity of fosgo-AM to augment this HGF-dependent behavior in colonies of MDCK cells. In this assay, colony cultures were stimulated for 24 h, during which, activated colonies spread into individual cells. This behavior was quantified by staining the cells with a nonspecific surface fluorescent stain (wheat germ agglutinin conjugated to CF488), imaging fields of cells, and comparing an automated count of the number of distinct objects normalized to the total area stained in an individual well. An HGF response standard curve was produced to identify a subthreshold concentration for cotreatment with fosgo-AM. Normalized particle counts increase with ascending doses of HGF, ultimately reaching significance with 40 ng/mL HGF (Fig. 2e). A subthreshold dose of HGF (10 ng/mL) was selected that is insufficient to produce significant colony-scattering behavior. HGF (10 ng/mL) was combined with fosgo-AM across a range of doses (Fig. 2f). A one-way ANOVA indicated a significant treatment effect (*F*(5,23) = 6.968, *p* = 0.004) and post hoc multiple comparisons suggested that fosgo-AM, in combination with HGF, resulted in significant cell scattering at doses of 10 pM, 100 pM, and 1 nM (*p* < 0.05), each compared with HGF treatment alone, suggesting that fosgo-AM drives HGF/MET-dependent cellular behaviors.

### Fosgo-AM Promotes Neurite Outgrowth and Synaptogenesis

**Fig. 3:**
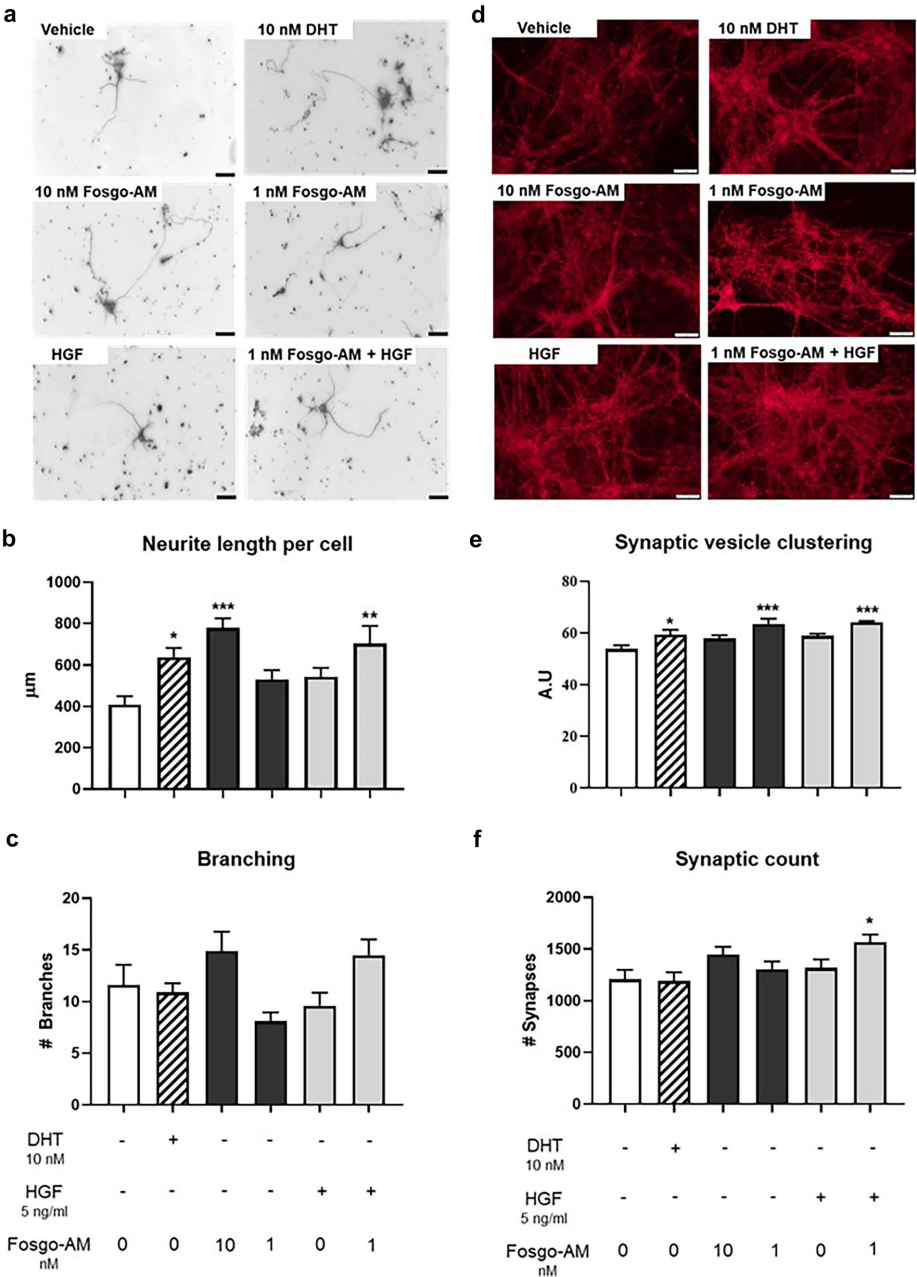
Fosgo-AM promotes neurite outgrowth and synaptogenesis. **a** Representative images depicting the effect of hepatocyte growth factor (HGF) ± fosgonimeton active metabolite (fosgo-AM) on neurite outgrowth via immunofluorescence staining. Primary rat hippocampal neurons were cultured and treated with HGF ± fosgo-AM for 4 days, and stained for beta-III tubulin, a neuron-specific microtubule marker. Dihydrotestosterone (DHT) used as positive control. Scale bar = 20 µm. **b** Quantification of neurite outgrowth. Fosgo-AM (10 nM) and fosgo-AM (1 nM) + HGF significantly increased neurite outgrowth compared with vehicle alone. **c** Quantification of dendritic branching. Images (*n* = 10) of single cells were analyzed using NeuroJ; neurite length and number of branches were calculated for each cell, and the average of each treatment group was compared with the average of the vehicle. **d** Representative images depicting the effect of HGF ± fosgo-AM on synaptogenesis via immunofluorescence staining. Primary rat hippocampal neurons were cultured and treated with HGF ± fosgo-AM for 8 days, and stained for synaptobrevin-II, a marker of synaptic vesicles. Scale bar = 20 µm. **e** Quantification of immunofluorescence of synaptobrevin-II intensity as an indirect measure of synaptic strength i.e., synaptic vesicle clustering. Both fosgo-AM (1 nM) ± HGF (5 ng/mL) significantly increased synaptobrevin-II fluorescence intensity compared with vehicle alone. **f** Quantification of synaptic count. The number of synapses was significantly increased when neurons were treated with fosgo-AM (1 nM) + HGF (5 ng/mL) compared with vehicle alone. Images (*n* = 10) of single cells were analyzed using NeuroJ; synaptic strength (relative abundance of presynaptic vesicles per synapse as measured by synaptobrevin-II fluorescence intensity) and synaptic count (number of synapses) were calculated for each cell, and the mean of each treatment group was compared with the average of the vehicle. Data shown are mean ± SEM. For neurite length per cell, statistical differences were determined by Kruskal–Wallis test followed by Dunn’s multiple comparison post-test. For all others, statistical differences were determined by one-way ANOVA followed by Dunnett’s multiple comparison post-test. *p < 0.05, **p < 0.01, ***p < 0.001 compared with vehicle

It is well-established that activation of the HGF/MET pathway can induce potent neurotrophic signaling leading to increased neuroplasticity [11]. Therefore, we sought to assess whether positive modulation of HGF/MET by fosgo-AM can mediate neurotrophic signaling to enhance neuronal development and synaptogenesis. To determine the effects of fosgo-AM treatment on neurite outgrowth, primary rat hippocampal neurons were cultured for 4 days and treated with HGF ± fosgo-AM on the first and third day of culture. At the end of the growth period, the cells were fixed and stained for beta-III tubulin, a neuron-specific microtubule marker, and assessed for neurite length and dendritic branching using immunofluorescence microscopy (Fig. 3a). A Kruskal–Wallis test indicated a significant treatment effect on neurite length per cell (*p* < 0.001) and post hoc multiple comparisons indicated that hippocampal neurons treated with 10 nM fosgo-AM exhibited a significant increase in neurite length per cell compared with vehicle (*p* < 0.001; Fig. 3b). Cotreatment with 1 nM fosgo-AM and 5 ng/mL HGF also significantly increased neurite length per cell (*p* < 0.01), whereas treatment with HGF alone showed no significant differences compared with vehicle (Fig. 3b). The number of neurite branches were also counted in these cultures, and a similar trend was observed, but this effect did not reach statistical significance (Fig. 3c). Although one-way ANOVA suggested a significant treatment effect on the number of branch points per cell (*F*(5, 54) = 3.373), *p* = 0.01), post hoc multiple comparisons did not detect any significant differences among the treatments.

To determine the effects of fosgo-AM on synaptogenesis, immunofluorescence intensity of synaptobrevin-II, a marker of synaptic vesicles, was evaluated in primary hippocampal neurons (Fig. 3d). Synaptobrevin-II staining intensity can be used as a metric of synaptic vesicle clustering, speaking to the density of vesicles available for propagating a signal, thus acting as an indirect measure of synaptic strength [27]). Cells were cultured for 8 days with treatment added on day 1 and refreshed on days 3 and 6. Synaptobrevin-II staining intensity (relative abundance of presynaptic vesicles per synapse) was quantified as an indicator of synaptic strength, and synaptic count was determined by quantifying the number of strongly labeled puncta. A one-way ANOVA indicated a significant treatment effect on both synaptobrevin-II staining intensity (*F*(5,54) = 6.688, *p* < 0.001) and synaptic count (*F*(5,54) = 3.196, *p* = 0.0134). Post hoc multiple comparisons indicated that treatment with 1 nM fosgo-AM (*p* < 0.001), and cotreatment of 1 nM fosgo-AM with 5 ng/mL HGF (*p* < 0.001) significantly enhanced immunofluorescence intensity of synaptobrevin-II compared with vehicle (Fig. 3e). In addition, cotreatment of 1 nM fosgo-AM and 5 ng/mL HGF significantly increased the number of synapses (*p* < 0.05) compared with vehicle (Fig. 3f). Collectively, these observations suggest that fosgo-AM can induce potent neurotrophic effects to facilitate development of neuronal projections and formation of new synapses.

### Fosgo-AM Is Neuroprotective in Primary Neuron Culture

**Fig. 4:**
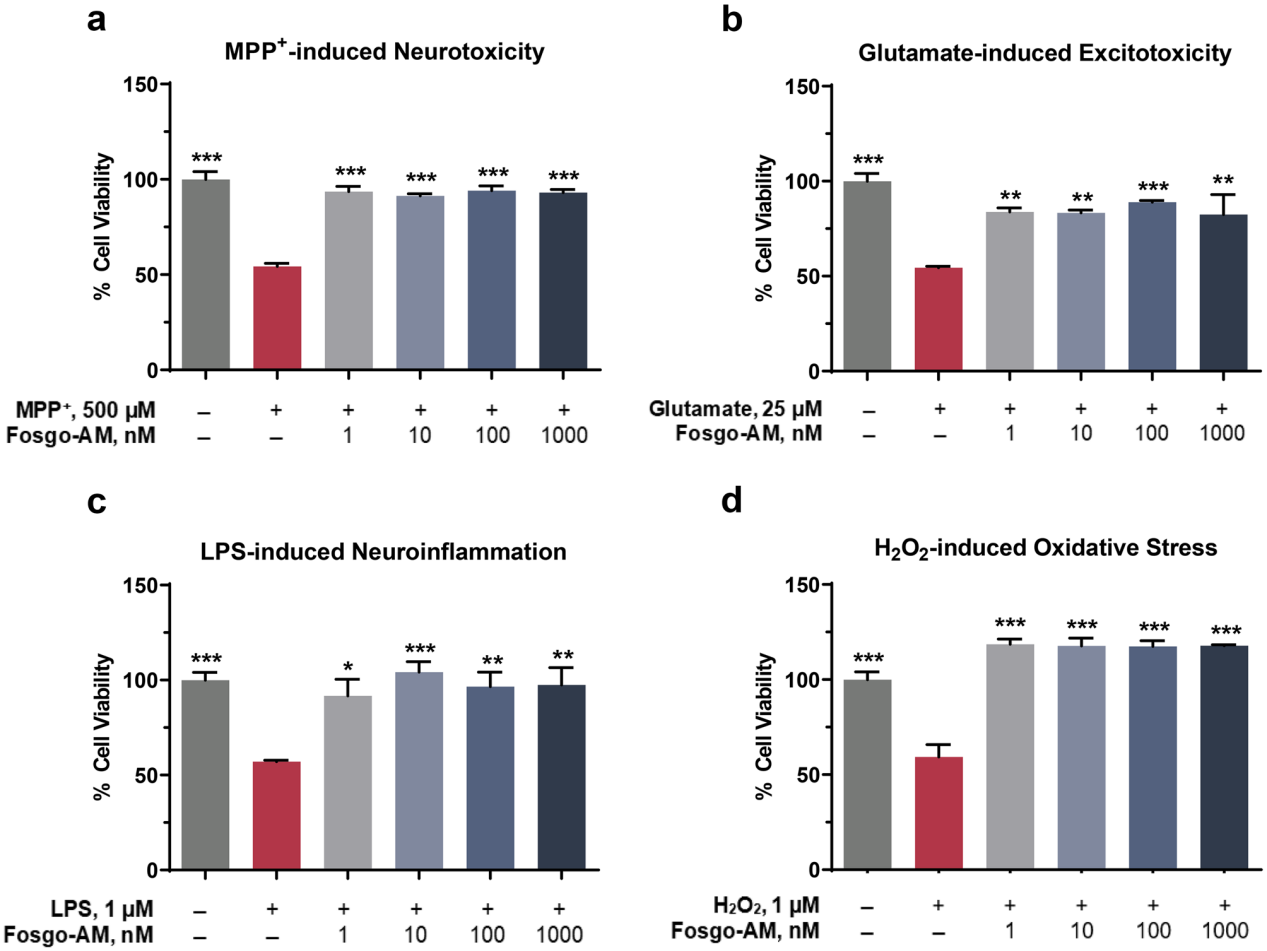
Fosgo-AM is neuroprotective in primary neuron culture. **a**–**d** Bar graphs depicting the percentage of cortical neurons surviving after exposure to several neurological insults in the presence and absence of fosgo-AM. Primary rat cortical neurons were treated with vehicle or fosgo-AM (1 nM, 10 nM, 100 nM, or 1000 nM) for 15 min, and subjected to one of the following neurotoxic compounds: **a** methyl-4-phenylpryidinium (MPP^+^; 500 µM), **b** glutamate (25 µM), **c** lipopolysaccharide (LPS; 1 µM), or **d** hydrogen peroxide (H_2_O_2_; 1 µM) for 24 h. Compared with the vehicle-treated group, cortical neurons pretreated with fosgo-AM (1 nM, 10 nM, 100 nM, or 1000 nM) exhibited significant improvement in cell viability against **a** MPP^+^, **b** glutamate, **c** LPS, or **d** H_2_O_2_. Data for each treatment expressed as a percentage of control (100% cell viability), averaged (*n* = 4–6) and presented as mean ± SEM. Statistical differences were determined by one-way ANOVA followed by Tukey's multiple comparison post-test. *p < 0.05, **p < 0.01, ***p < 0.001 compared with insult

Activation of the HGF/MET system has been shown to counteract neuronal damage induced by glutamate toxicity, oxidative stress, and ischemic injury [17, 28–31]. HGF/MET signaling exerts antiapoptotic effects via activation of ERK and/or AKT and supports expression of neuroprotective genes *Bcl-2* and *Bcl-xl* [32]. Therefore, we investigated whether positive modulation of HGF/MET by fosgo-AM can mediate neuroprotective effects in primary neuron culture. Mature rat cortical neurons were treated with fosgo-AM and subjected to several neurotoxic compounds, including 1-methyl-4-phenylpryidinium (MPP^+^), glutamate, lipopolysaccharide (LPS), or hydrogen peroxide (H_2_O_2_) for 24 h. Neuronal survival was then assessed using a Cell Titer-Glo (CTG) assay to determine degree of cell viability relative to control. One-way ANOVA indicated a significant treatment effect on cell viability in all 4 neurotoxicity assays: MPP^+^ (*F*(5,20) = 30.93, *p* < 0.001), glutamate (*F(*5,20) = 10.74, *p* < 0.001), LPS (*F*(5,20) = 6.908, *p* = 0.0007), and H_2_O_2_ (*F*(5,20) = 31.82, *p* < 0.001). Post hoc analyses indicated that cortical neurons treated with fosgo-AM exhibited significant improvement in cell viability against neuronal damage induced by MPP^+^ (Fig. 4a), glutamate (Fig. 4b), LPS (Fig. 4c), and H_2_O_2_ (Fig. 4d). For example, treatment with 1 µM fosgo-AM rescued cell viability against neurological insults with the following percentages of recovery: 85% versus MPP^+^, 61% versus glutamate, 93% versus LPS, and 144% versus H_2_O_2_. Significant improvement in cell viability was also observed at all tested concentrations of fosgo-AM against all insults, highlighting its potent neuroprotective actions.

### Fosgo-AM Rescues Scopolamine-Induced Learning Deficits

Administration of the cholinergic receptor antagonist scopolamine significantly impairs spatial learning, producing a deficit in Morris water maze performance in rodents [33]. Previous studies have shown that activation of NMDA receptors can reverse these scopolamine-induced cognitive deficits [34–36], and HGF signaling is known to promote NMDA-receptor activation [15]. Furthermore, overexpression of HGF in the CNS of rodents results in improved performance in the Morris water maze, likely mediated in part through NMDA-receptor modulation [37]. These studies led us to hypothesize that fosgo-AM treatment would be effective in reversing the cognitive deficits in the preclinical scopolamine model of dementia.

**Fig. 5:**
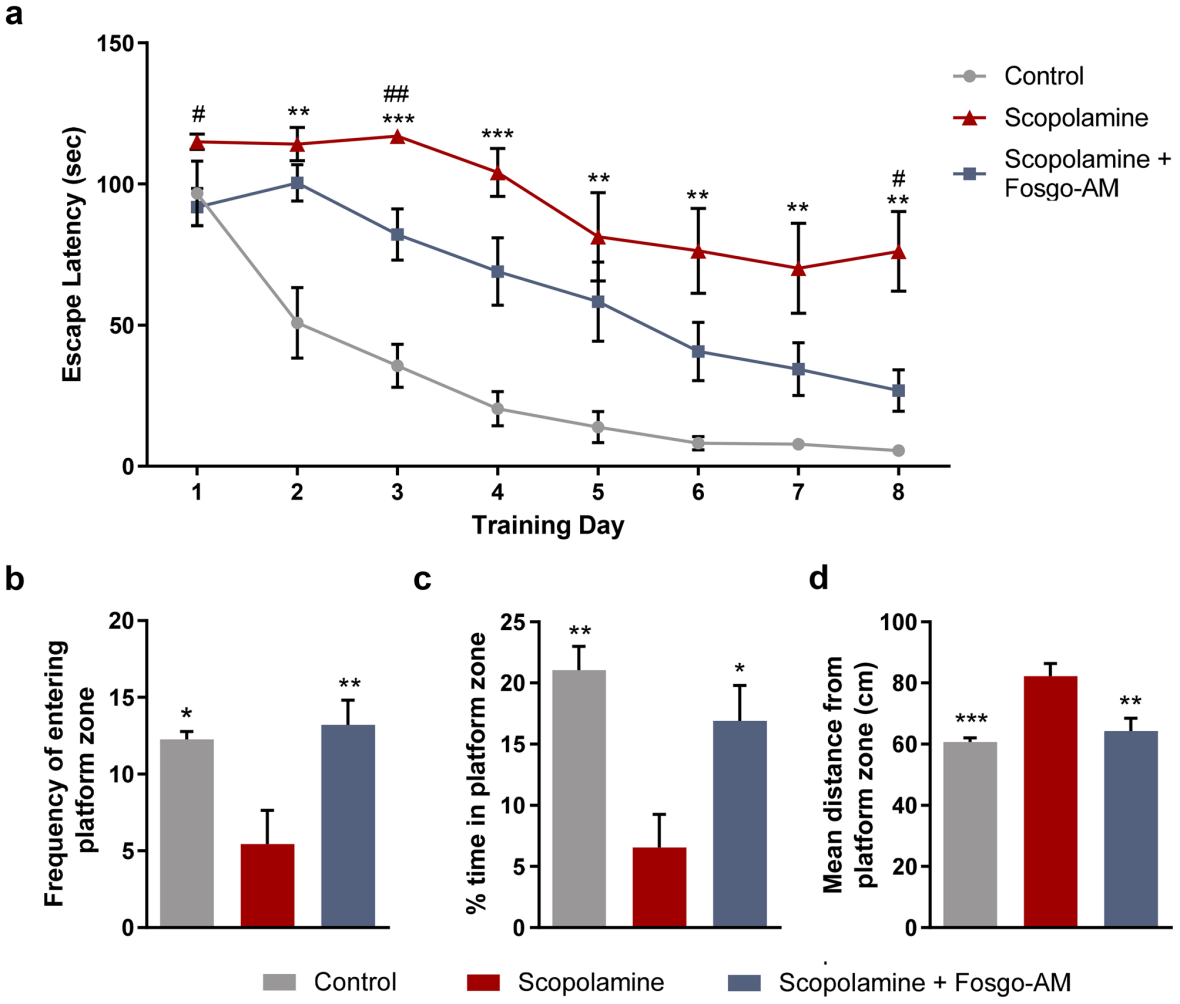
Fosgo-AM reverses scopolamine-induced spatial learning deficits. **a** Group latencies to find the submerged platform in the Morris water maze task of spatial memory are shown. On each of the 8 days of water maze acquisition, rats were treated with either fosgonimeton active metabolite (fosgo-AM) (1 mg/kg) or vehicle via subcutaneous injection, followed by scopolamine (105 nmol in 4 μL of aCSF) via intracerebroventricular injection. The learning curve for fosgo-AM–treated animals was significantly different from animals in the vehicle group. Twenty-four hours after the final acquisition day, a single probe trial was performed to measure learning persistence by removing the platform and allowing the animals to freely swim the maze for 120 s. **b** The frequency of entering the virtually defined platform zone, **c** percentage of time spent in the platform zone, and **d** mean distance from the center of the platform were calculated for each animal. Treatment with fosgo-AM significantly restored all measures of learning persistence compared with scopolamine + vehicle. Statistical differences in escape latency were determined by two-way ANOVA with Dunnett’s post hoc test. **p < 0.01, ***p < 0.001 for the control group and ^#^*p* < 0.05, ^##^*p* < 0.01 for the treated group compared with the scopolamine group. Statistical differences in performance on probe trial were determined via one-way ANOVA with a Bonferroni post hoc test. *p < 0.05, **p < 0.01, ***p < 0.001. Data shown are mean ± SEM; *n* = 8 for control group, *n* = 9 for scopolamine group, and *n* = 10 for scopolamine + fosgo-AM group

Control rats not receiving scopolamine significantly improved performance in the water maze over the 8-day training period, indicating successful learning of the task (F(7, 105) = 4.05, *p <* 0.001).  In comparison, rats receiving scopolamine in the absence of fosgo-AM showed poor performance in the Morris water maze with little improvement in escape latency over the course of the acquisition phase. SC delivery of 1 mg/kg fosgo-AM led to reduced escape latency over time, suggesting a rescue of the scopolamine-induced learning deficits (Fig. 5a). Additionally, fosgo-AM significantly improved the frequency of entering the platform zone (*F*(2, 24) = 6.54, *p* < 0.001), percentage of time spent in the platform zone (*F*(2, 24) = 7.83, *p* < 0.01) and mean distance from the center of the platform (*F*(2, 24) = 9.68, *p* < 0.001) compared with vehicle-treated scopolamine animals in the probe trial conducted 24 h after training completion (Fig. 5b–d). In these measures, animals that received scopolamine + fosgo-AM demonstrated successful retention of the water maze task. Taken together, these data indicate that fosgo-AM, when delivered subcutaneously, is neuroactive and significantly improves spatial learning deficits from a scopolamine challenge.

### The Prodrug Fosgonimeton Improves Fosgo-AM PK

Fosgo-AM has excellent potency and successfully reversed cognitive deficits in vivo, which presented opportunities for further refinement of its physiochemical properties for further clinical development. Fosgo-AM has poor stability when exposed to the digestive enzymes present in gastric and intestinal fluids, with only 7.8% and 0.1% remaining after 1 h of exposure to simulated gastric fluid and intestinal fluid, respectively, and ultimately has poor oral bioavailability with high variability in exposure. These results suggest that fosgo-AM is likely not suitable as an oral drug. Other routes of administration were explored for fosgo-AM; however, the maximum aqueous solubility of fosgo-AM is less than 50 μg/mL, which limits the use of other clinically viable routes, including SC injection. In vivo activity studies using fosgo-AM required a pure DMSO vehicle to achieve reasonable dose volumes.

**Fig. 6:**
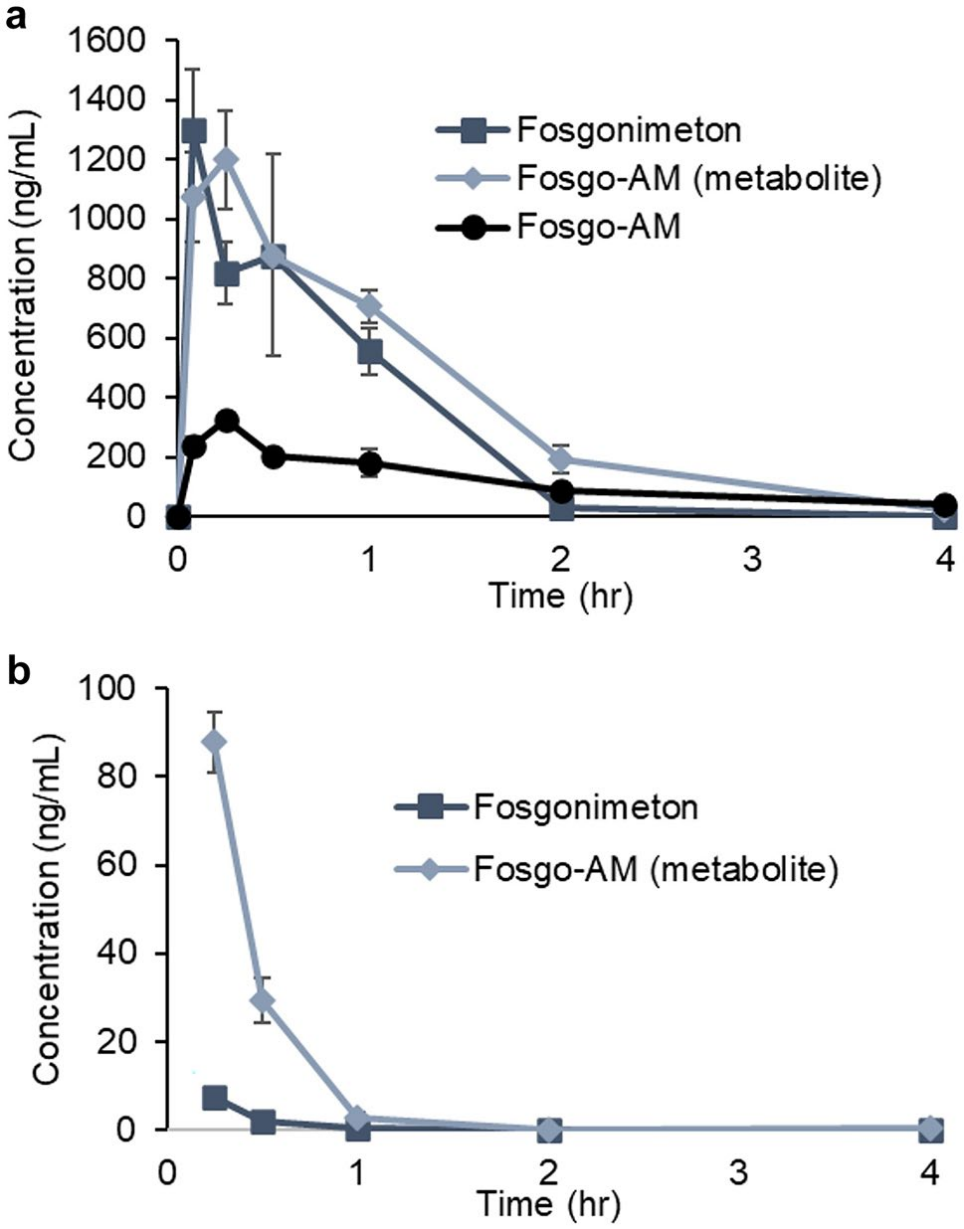
Fosgonimeton is rapidly converted in vivo resulting in higher exposure of the active metabolite, fosgo-AM. Fosgonimeton is rapidly metabolized to the active metabolite fosgo-AM after SC delivery (here labeled fosgo-AM [metabolite]) and results in higher exposure than the equivalent SC dose of fosgo-AM directly (labeled fosgo-AM). **a** Plasma exposure of fosgo-AM in rats after SC delivery of fosgo-AM (10 mg/kg, black circle) versus the plasma exposure of fosgonimeton and fosgo-AM (metabolite) after SC delivery of the equivalent dose of fosgonimeton (12 mg/kg, blue square and diamond, respectively). **b** Plasma exposure of fosgonimeton and fosgo-AM (metabolite) in mice after SC delivery of 0.75 mg/kg fosgonimeton showing rapid conversion of the prodrug to the parent compound. Data are presented as mean ± SEM; rat PK studies *n* = 4, mouse PK study *n* = 3

To address the solubility limitations of fosgo-AM and enable SC delivery, a prodrug strategy was used. A series of prodrugs were synthesized, and their relative solubility was evaluated. This led to the development of fosgonimeton, which is soluble at concentrations greater than 70 mg/mL in aqueous solutions. PK studies were conducted to evaluate metabolism of fosgonimeton to fosgo-AM in vivo and to monitor exposure of both compounds (Fig. 6). After SC delivery, fosgonimeton was rapidly metabolized to fosgo-AM, with the active compound appearing by the first tested time points: 5 min in rats and 15 min in mice. Delivery of fosgonimeton results in higher systemic exposure and a nearly 4-fold higher maximum concentration (*C*_max_) in rats compared with the equivalent direct dose of fosgo-AM, with a fosgo-AM concentration of 1198 ng/mL after delivery of fosgonimeton, after metabolism, and only 329 mg/mL after direct delivery of fosgo-AM. CNS penetration and distribution of fosgonimeton and its active metabolite fosgo-AM were measured after SC injection of fosgonimeton (Table 1). Fosgo-AM distributed to all regions with a time to maximum concentration (*T*_max_) of 10 min and an exposure range from 23.75 to 58.30 ng/g of tissue. Fosgonimeton was undetectable in all brain regions, suggesting it did not penetrate the blood–brain barrier. CNS activity after delivery of fosgonimeton is therefore dependent on the activity of its metabolite fosgo-AM. Fosgonimeton physiochemical characteristics are substantially improved compared with fosgo-AM, resulting in superior PK properties, and fosgonimeton was selected for further development.

**Table 1:** The active metabolite fosgo-AM, and not the prodrug fosgonimeton, distributes to the CNS. Male Sprague Dawley rats were dosed with 12 mg/kg fosgonimeton, SC. At each time point (0.16, 0.5, and 1 h after dosing), perfused brains were collected and dissected into individual regions. The concentrations of fosgonimeton and its metabolite fosgo-AM were analyzed in each region. Fosgo-AM was found in all brain regions analyzed, whereas fosgonimeton was not detected in any brain region; *n* = 3. Additionally, distributions of fosgonimeton and fosgo-AM in the whole brain were assessed in male and female C57BL/6 mice at 0.16 h after a 5-mg/kg IV dose. Fosgo-AM was detectable in mouse brain, whereas fosgonimeton was not detected; *n* = 4 (2 male, 2 female)

**Tissue**	***C***_**max**_**, ng/g ± SEM**		***T***_**max**_**, hours**	
	**Fosgonimeton**	**Fosgo-AM (metabolite)**	**Fosgonimeton**	**Fosgo-AM (metabolite)**
**Rats, 12 mg/kg SC**				
Whole brain	ND	31.9 ± 23.8	ND	0.16
Striatum	ND	24.1 ± 12.8	ND	0.16
Olfactory bulb	ND	43.7 ± 7.3	ND	0.16
Hippocampus	ND	49.8 ± 30.5	ND	0.16
Cerebellum	ND	23.8 ± 4.1	ND	0.16
Cerebral cortex	ND	36.5 ± 12.0	ND	0.16
Brainstem	ND	58.3 ± 11.3	ND	0.16
**Mice (5 mg/kg IV)**				
Whole brain	ND	7.9 ± 2.3	NT	NT

*IV* intravenous, *ND* not detected, *NT* not tested, *SC* subcutaneous, *SEM* standard error of the mean

### Fosgonimeton Prevents LPS-Induced Cognitive Deficits In Vivo

**Fig. 7:**
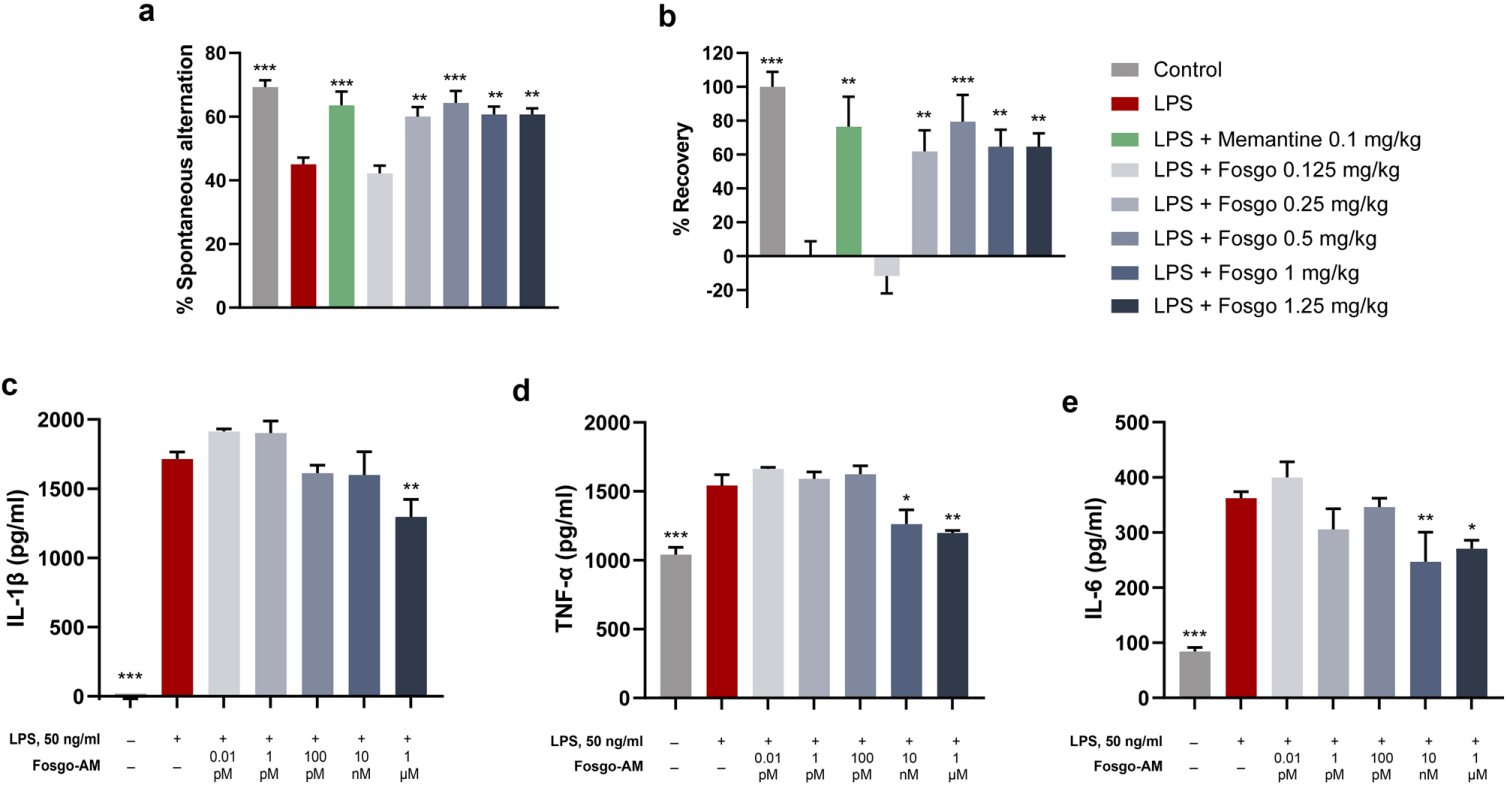
Fosgonimeton prevents LPS-induced cognitive deficits in vivo. **a** Percentage of spontaneous alternations in the T-maze are shown. Mice were injected with lipopolysaccharide (LPS; 0.25 mg/kg, I.P.) at day 1, then received daily treatment with fosgonimeton, vehicle, or positive control memantine for 14 days. On day 14, mice were assessed for performance in the spontaneous alternation T-maze task. Memantine (0.1 mg/kg, I.P.), as well as fosgonimeton at 0.25, 0.5, 1, and 1.25 mg/kg S.C., significantly prevented LPS-induced cognitive deficits. Data presented as mean ± SEM; *n* = 10. **b** Percentage recovery in spontaneous alternations is shown by group. Percentage recovery was significantly increased with memantine, as well as with fosgonimeton at 0.25, 0.5, 1, and 1.25 mg/kg. Data presented as mean ± SEM; *n* = 10. **c**–**e** Bar graphs showing the levels of LPS-induced interleukin 1 beta (IL-1β), tumor necrosis factor α (TNF-α), and IL-6 release in THP-1 differentiated macrophages in the presence and absence of fosgo-AM at 0.01 pM, 1 pM, 100 pM, 10 nM, or 1 µM. Compared with their respective vehicle-treated group, THP-1 differentiated macrophages treated with fosgo-AM (1 µM) exhibited a significant reduction in release of **c** IL-1β, **d** TNF-α, and **e** IL-6 Data for each treatment were averaged and presented as mean ± SEM; Number of experimental replicates was as follows: *n* = 6 for LPS + vehicle for all 3 cytokine assays, *n* = 3 for LPS + fosgo-AM for all 3 cytokine assays; for vehicle groups, *n* = 3 (IL-1β assay), 5 (TNF-α assay), and 6 (IL-6 assay). Statistical differences in percentage of spontaneous alterations were determined by one-way ANOVA with Dunnett’s post hoc test. Statistical differences in percentage of recovery were determined by Kruskal–Wallis test with Dunn’s post hoc test. Statistical differences in IL-1β, TNF-α, and IL-6 were determined by one-way ANOVA followed by Dunnett’s multiple comparison post hoc test *p < 0.05, **p < 0.01, ***p < 0.001 compared with LPS only

In vitro neuroprotection studies using an LPS challenge illuminated a potential anti-inflammatory effect of positively modulating HGF/MET signaling via fosgo-AM. Neuroinflammation is recognized as a critical component of neurodegenerative disease leading to dementia [38]. To assess the functional benefits of its anti-inflammatory effects, we evaluated fosgonimeton in an LPS-induced model of cognitive impairment in mice. Healthy, non–cognitively impaired rodents preferentially explore novel environments. In the T-maze apparatus, novelty seeking manifests as alternation behavior, that is, exploring open arms in a sequential (e.g., left–right–left–right) rather than repetitive (e.g., left–left–right–right) order [39]. Alternation behaviors in a T-maze were assessed 2 weeks after a single, acute, intraperitoneal injection of LPS. The LPS challenge resulted in significant cognitive impairment, quantified as a reduction in percentage of spontaneous alternations compared with mice receiving the saline vehicle (*F*(8, 81) = 0.80, *p* < 0.001). Two consecutive weeks of once-daily fosgonimeton SC significantly ameliorated these deficits at all doses tested (*F*(8, 81) = 0.80, *p* < 0.01 for 0.25 mg/kg, 1 mg/kg, 1.25 mg/kg; *p* < 0.001 for 0.5 mg/kg), except for the lowest dose tested, 0.125 mg/kg (*p* > 0.05) (Fig. 7a). To quantify the level of recovery relative to the deficit of the model, percentage of recovery was calculated at 61.76% for the 0.25mg/kg treated group, 79.40% for the 0.5mg/kg treated group, 64.70% for the 1mg/kg treated group, and 64.70% for the 1.25mg/kg (Fig. 7b). In comparison, the positive control, NMDA receptor antagonist memantine, had a percentage of recovery of 76.40%. These data indicate that fosgonimeton has procognitive properties.

To further investigate the mechanism by which positive modulation of HGF/MET by fosgonimeton may counteract LPS-induced deficits, we sought to assess its effects on LPS-induced release of IL-1β, TNF- α, and IL-6 in vitro. These cytokines are widely implicated in CNS pathophysiological processes, including synaptic dysfunction and neuroinflammation [40–45]. Fosgonimeton, as a prodrug, requires an in vivo environment to ensure optimal conversion to its active metabolite, fosgo-AM. Therefore, fosgo-AM is used for in vitro experiments. To determine the effect of fosgo-AM on cytokine release, THP-1 monocytes were differentiated into macrophages and subjected to LPS treatment in the presence and absence of fosgo-AM, and assayed for levels of IL-1β, TNF- α, or IL-6 in the cell culture supernatant. One-way ANOVA revealed a significant treatment effect on levels of IL-1β (*F*(6, 16) = 56.47, *p* < 0.001), TNF-α (*F*(6,19) = 12.97, *p* < 0.001), and IL-6 (*F*(6,20) = 28.29, *p* < 0.001). Post hoc multiple comparisons indicated that LPS-challenged THP-1 macrophages treated with 1 µM fosgo-AM exhibited a significant decrease in levels of IL-1β release (*p* < 0.01; Fig. 7c), TNF-α release (*p* < 0.01; Fig. 7d), and IL-6 release (*p* < 0.05; Fig. 7e) compared with their respective LPS-challenged vehicle group.

## Discussion

Overall, our findings provide evidence that the small-molecule fosgonimeton, via its active metabolite fosgo-AM, may provide therapeutic benefit by positively modulating HGF/MET signaling in dementia and other types of cognitive impairment that involve neurodegeneration and synaptic loss, mitochondrial dysfunction, excitotoxicity, oxidative stress, cholinergic deficits, and neuroinflammation. In addition, the reported studies highlight the ability of fosgonimeton to improve learning and memory in preclinical models of cognitive impairment.

We identified a series of small-molecule positive modulators of HGF/MET, including fosgo-AM, which show significant activity in the picomolar range. For these highly potent compounds, the full hormetic dose–response curve can be seen in the low picomolar to high nanomolar range tested. Hormetic dose–response curves are used to describe compounds that are inactive at high and low doses while having peak activity at intermediate doses. This is common in hormone and growth factor–based systems; HGF-induced activation of MET also displays a hormetic dose–response curve [46–48]. The appearance of a hormetic dose–response curve suggests that compounds in this class, including fosgo-AM, are functioning within the normal regulatory systems surrounding HGF/MET activation.

The most potent positive modulator of HGF/MET identified, fosgo-AM, significantly enhanced activation of ERK and AKT pathways in the presence of a subthreshold concentration of HGF, implying that positive modulation of HGF/MET by fosgo-AM is sufficient to activate the entire downstream HGF/MET pathway. Furthermore, MDCK cell scattering, known to be activated by MET [24–26], was enhanced by treatment with fosgo-AM, confirming that the activation of MET and its downstream effectors can have effect on cellular behaviors. The assays for pMET, activation of downstream signaling molecules, and cellular behaviors had different sensitivities to HGF itself and to fosgo-AM. The cause of these variations is unknown but might reflect differential MET expression or activity between cell types, levels of background signaling, or sensitivity of assays themselves, as evidenced by the differences in HGF sensitivity. Despite these differences in sensitivity, fosgo-AM reliably modulated the HGF/MET pathway, indicating that the mechanism of action of fosgonimeton is through positive modulation of this system.

Fundamentally, dementia arising from neurodegenerative disorders is driven at least in part by loss of synaptic connections [49–52]. We have demonstrated that treatment with fosgo-AM significantly enhanced synaptogenesis, synaptic vesicle clustering (a surrogate metric of synaptic strength), and induced neurotrophic effects similar to those of exogenous HGF treatment in disease-relevant tissues [11, 12].

Dementia is also associated with a variety of pathophysiological facets, including mitochondrial dysfunction, excitotoxicity, neuroinflammation, and oxidative stress [4, 5]. Herein, we provided evidence that positive modulation of HGF/MET by fosgo-AM confers protection to cortical neurons challenged with neurotoxic insults that mimic critical aspects of neurodegeneration: MPP^+^, glutamate, LPS, and H_2_O_2_. MPP^+^ is a potent inhibitor of mitochondria complex I that induces ATP depletion, breakdown of the electron transport chain, generation of reactive oxidative species (ROS), caspase-3 activation, and subsequent apoptosis [53, 54]. Glutamate excitotoxicity is a pathological hallmark of neurodegeneration in which excessive and prolonged activation of glutamatergic signaling leads to intracellular Ca^2+^ overload, loss of mitochondrial membrane potential, oxidative stress, and eventually cell death [55]. LPS is a potent activator of toll-like receptor 4 (TLR4), which is expressed on neurons and microglia, and can initiate a series of proinflammatory pathways. For example, LPS induces strong release of TNF-α and IL-6, both of which are key proinflammatory mediators that lead to neuronal damage [56, 57]. H_2_O_2_ is a significant source of free radicals, directly elevating levels of ROS [58]. These neurodegenerative components are not mutually exclusive. Instead, they exist in a positive feedback loop that creates an ongoing cycle of mitochondrial damage, ROS production, neuroinflammation, excitotoxicity, and neuronal damage. In this regard, the ability of fosgo-AM to protect neurons from all tested insults highlights its ability to not only directly mitigate several cellular stressors, but also potentially interrupt the neurodegenerative cascade as a whole. Although the molecular mechanisms downstream of HGF/MET by which fosgo-AM confers protection to these various insults are not yet elucidated, a plausible explanation may lie in its ability to activate prosurvival signaling cascades mediated by ERK and/or AKT that promote induction of neuroprotective genes to counteract cell death.

The in vivo effects of fosgo-AM were first evaluated in the scopolamine-induced amnestic model of dementia in the Morris water maze. Scopolamine, a muscarinic receptor antagonist, blocks the cholinergic neurotransmitter system in animal models and produces learning and memory deficits similar to the cognitive dysfunction reported in AD and other dementias [36, 59, 60]. Rats treated with fosgo-AM showed significant improvement in learning acquisition and memory retention in this test compared with those treated with vehicle alone. These results are consistent with previous reports that enhancement of HGF/MET activity boosts learning and memory performance in mice [37]. These effects may be at least partially mediated by the potentiation of NMDA receptor signaling [37]. HGF treatment of neuronal cultures has been shown to modulate NMDA receptor activity through activation of effector proteins, including protein kinase C [15, 61], which phosphorylates NR1 subunits of the NMDA receptor and modulates surface expression, synaptic localization, and activation of the NMDA receptor [15, 62, 63]. Increased synaptic localization of the NMDA receptor [14, 64] and augmentation of transreceptor currents may bolster LTP and related memory formation [15, 65]. In this way, potentiation of NMDA receptors by positive modulators of HGF/MET may offset the cognitive impairment induced by cholinergic deficits seen in the scopolamine model; however, this warrants further exploration.

Although fosgo-AM demonstrated significant neurotrophic and neuroprotective effects in vitro and procognitive effects in vivo, its physiochemical properties presented hurdles to further development. To this end, fosgonimeton, a prodrug of fosgo-AM, was developed to allow for reliable delivery in clinically viable formulations and routes of administration. Delivery of fosgonimeton via SC administration leads to increased exposure of fosgo-AM compared with the equivalent direct dose of fosgo-AM, suggesting a lower dose of fosgonimeton might be used to achieve similar exposures of fosgo-AM. This property may have several advantages, including the possibility of exploring larger dose ranges and minimizing dose volume. Fosgonimeton does not penetrate the blood–brain barrier until it is converted into fosgo-AM; thus, the procognitive effects of the prodrug fosgonimeton are attributed to the effects of its active metabolite fosgo-AM.

To confirm that fosgonimeton can convert to fosgo-AM, distribute to the brain, and subsequently confer procognitive effects in vivo, we assessed the ability of fosgonimeton treatment to prevent cognitive impairment in an LPS-induced neuroinflammation model in mice. LPS administration induces an acute systemic inflammatory reaction that triggers a prolonged neurodegenerative response that includes synaptic loss, amyloid β deposition, and neuronal death, ultimately resulting in cognitive impairment [66, 67]. As anticipated, we observed clear LPS-induced cognitive impairment in mice using the T-maze spatial memory test, and treatment with fosgonimeton attenuated these deficits at multiple doses. The level of rescue observed in the best dose of fosgonimeton was comparable in this model with that of the approved AD therapeutic memantine.

In LPS-challenged THP-1 macrophages, fosgo-AM significantly reduces the release of IL-1β, TNF-α, and IL-6, all of which are key mediators of CNS inflammatory responses [40–45]. Such observations provide a potential mechanism by which fosgonimeton reverses LPS-induced cognitive impairment.

Based on the known neurotrophic effects of the HGF/MET system and the findings presented here, positive modulators of HGF/MET activity, such as fosgonimeton, could be expected to produce a multitude of outcomes. Multiple lines of evidence indicate that activation of the HGF/MET system (1) stimulates neural stem cell proliferation, differentiation, and neurogenesis; (2) induces neurite outgrowth and synaptogenesis; (3) supports healthy neuronal networks; (4) promotes cell survival and resistance to damage; (5) modulates neuroinflammation; and (6) promotes clustering and activity of NMDA receptors at the synaptic cleft to enhance procognitive NMDA receptor activity, including LTP, while preventing excitotoxicity [11, 13, 14, 16, 31, 64, 68]. These effects may directly impact the neurodegeneration, reduced neuroplasticity, and neurotoxic environment seen in AD and other types of dementia. Treatment with fosgonimeton or the active metabolite fosgo-AM markedly improves cognitive performance in two independent models of cognitive impairment: the scopolamine rat and the LPS mouse models. Altogether, the findings presented here suggest that fosgonimeton can protect against several common pathophysiologies seen in dementia and justify further investigation into the therapeutic potential of fosgonimeton in patients with neurodegenerative disorders, including AD and other types of dementia.

## Supplementary Information

Below is the link to the electronic supplementary material.

Supplementary file1 (PDF 508 kb)

Supplementary file2 (PDF 440 kb)

Supplementary file3 (PDF 517 kb)

Supplementary file4 (PDF 434 kb)

Supplementary file5 (PDF 516 kb)

Supplementary file6 (PDF 508 kb)

Supplementary file7 (PDF 499 kb)

Supplementary file8 (PDF 517 kb)

Supplementary file9 (PDF 508 kb)

Supplementary file10 (PDF 508 kb)
